# Preparation of Pt and bamboo charcoal co-modified TiO_2_ for formaldehyde sensing at room temperature

**DOI:** 10.1098/rsos.231216

**Published:** 2024-07-03

**Authors:** Jian-Wei Lin, Yu-Xuan Wang, Hao Xu, Li-Zhu Huo, Xue-Juan Yang, Xi-Ping Luo

**Affiliations:** ^1^ Zhejiang Provincial Key Laboratory of Chemical Utilization of Forestry Biomass, College of Chemistry and Materials Engineering, Zhejiang A & F University, Hangzhou 311300, People’s Republic of China

**Keywords:** platinum-modified TiO_2_, nanostructured TiO_2_, bamboo charcoal, formaldehyde sensing, gas sensors

## Abstract

Anatase TiO_2_ has evolved into one of the most attractive materials for gas sensing owing to its strong oxidation activity and excellent sensing properties. In this study, we prepared Pt and bamboo charcoal co-modified nano-TiO_2_ using a one-pot hydrothermal process and applied it to detect formaldehyde. The successful incorporation of the precious metal Pt and bamboo charcoal onto TiO_2_ was confirmed by scanning electron microscope, transmission electron microscopy, energy dispersive spectrometer, X-ray diffraction and X-ray photoelectron spectroscopy. Detailed analysis revealed a homogeneous distribution of Pt nanoparticles and bamboo charcoal on the TiO_2_ surface, which significantly improved the surface area and facilitated gas adsorption. These modifiers significantly enhanced the response of TiO_2_ to formaldehyde, for instance, the response signal increased fourfold, while the response time decreased from 91 to 68 s. The sample with 0.5@Pt and 0.5@C bamboo charcoal performed the best, showcasing the synergistic effect of metal nanoparticles and carbonaceous materials on gas-sensing properties. Our work highlighted the potential of using biomass-derived carbon to enhance the detection of formaldehyde and demonstrated the importance of material characteristics in designing effective gas sensors.

## 1. Introduction

Formaldehyde, released from furniture and textiles in homes, is harmful to the human body [1]. It can cause irritation to the eyes and nose, damage the central nervous system and the immune system and lead to respiratory diseases and even blindness [2]. As a colourless irritant gas, formaldehyde is difficult to detect. Thus, it is crucial to develop simple detection methods for in-house applications. Among the various formaldehyde detection methods, such as liquid chromatography [3,4], spectroscopy [5–8], enzyme electrodes [9,10] and gas sensors [11–19], gas sensors are notable for being portable, inexpensive and capable of real-time monitoring of formaldehyde gas. So far, metal oxide-based semiconductors such as TiO_2_ [12], SnO_2_ [13,14], ZnO [15], WO_3_ [16–19] and In_2_O_3_ [20–22] have been extensively studied for the detection of formaldehyde gas owing to their high sensitivity, short response time and low cost in gas sensing. Among them, anatase TiO_2_, known for its low cost and non-toxicity, has been most commonly adopted [23]. However, the operating temperature of TiO_2_ sensors is usually high, limiting their applicability in general scenarios [24]. To enhance their formaldehyde sensitivity and reduce the operating temperature, noble metals such as Pt and Ag, along with metal oxides, have been widely adopted to modify the surface of TiO_2_. This, however, has inevitably increased the cost of the sensors [25]. Therefore, the search for low-cost alternative materials has become a significant task for the research community.

Bamboo, as a renewable resource, is widely available in China and many other countries. Bamboo charcoal, a solid material produced by the high-temperature carbonization of bamboo powder, offers a tubular carbon structure with high porosity, stability and electronic conductivity [26]. Its high specific surface area renders it a potent candidate for pollutant adsorption. For instance, Liao *et al*. discovered that bamboo charcoal exhibited a strong adsorption affinity for antibiotics, such as tetracycline and chloramphenicol [27]. Furthermore, Peng *et al*. successfully prepared bamboo-based activated carbon (BAC) from bamboo waste and enhanced its adsorption capacity through magnetization and modification, resulting in a novel amine-functionalized magnetic BAC [28].

Inspired by the excellent adsorption capability of bamboo charcoal, we have used it to co-modify TiO_2_ with Pt, aiming to improve the adsorption of formaldehyde onto the sensor. The bamboo charcoal-modified TiO_2_ (named TiO_2_/C) shows performance comparable to that of Pt/TiO_2_, while the Pt and bamboo charcoal co-modified TiO_2_ (named Pt/TiO_2_/C) exhibits a formaldehyde detection signal four times higher than that of unmodified TiO_2_ at room temperature.

## 2. Material and methods

### 2.1. Sample preparation

**Scheme 1.**
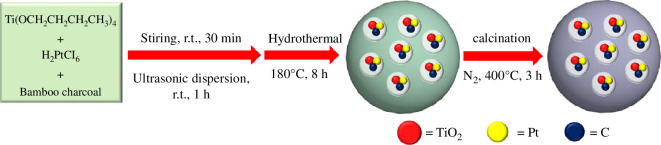
Schematic illustration of the Pt and bamboo charcoal co-modified TiO_2_ (Pt/TiO_2_/C) preparation process.

Nano-TiO_2_ was prepared via a hydrothermal method [29], as illustrated in scheme 1. The samples were prepared via a hydrothermal method for the following four reasons: (i) the hydrothermal method can produce high-quality nano-TiO_2_ materials, whose crystal morphology and crystal structure can be effectively controlled and adjusted; (ii) it is simple to operate, does not require high-end equipment and is suitable for large-scale production; (iii) nano-TiO_2_ materials synthesized by the hydrothermal method have good thermal stability and photocatalytic performance; and (iv) this method can use inexpensive precursors and solvents to prepare nano-TiO_2_ materials, which is cost-effective. Initially, 1 ml of tetra-butyl titanate was mixed with 15 ml of chloroplatinic acid solution, and 0.1 g of bamboo charcoal was added to the mixture. This mixture was stirred for 30 min using a magnetic stirrer and then subjected to an ultrasonic dispersing machine for 1 h. The sonicated solution was transferred into a Teflon-lined reactor and heated at 180°C for 8 h. After the reaction, the samples were collected by centrifugation, dispersed in distilled water and ethanol and then dried in an oven. The dried samples were subsequently heated in a tube furnace under N_2_ at 400°C for 3 h. Following the reaction, the samples were allowed to naturally cool to room temperature. Finally, the calcined samples were finely ground using an agate mortar to obtain the Pt and bamboo charcoal co-modified TiO_2_ (Pt/TiO_2_/C), with a molar ratio of Pt, TiO_2_ and C being 0.1 : 1 : 2.5.

### 2.2. Morphology characterization

X-ray diffraction (XRD) analysis was carried out using a Bruker AXS GmbH D2 Phaser X-ray diffractometer with Cu Kα radiation (*λ* = 1.5418 Å) in the 2θ range of 20°–80°. The operating voltage and current were 30 kV and 30 mA, respectively. The morphology and size of the products were observed using a cold-field emission scanning electron microscope (Hitachi SU8010) with an operating voltage of 15 kV. The morphology of the samples was observed using transmission electron microscopy (TEM, FEI Talos F200S) at 20 kV. The chemical state of the elements was analysed by X-ray photoelectron spectroscopy (XPS, Thermo Scientific K-Alpha) with an operating power of 72 W and an Al Kα excitation source.

### 2.3. Sensor performance testing

**Figure 1.**
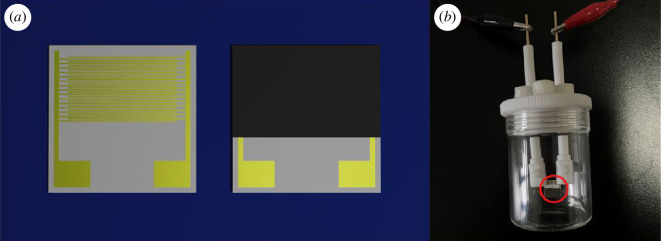
The picture of (*a*) the unmodified (left) and modified (right) interdigital electrode array and (*b*) gas detection device.


For electrical and sensing characterizations, a small amount of Pt and bamboo charcoal co-modified TiO_2_ obtained by grinding was mixed with distilled water and stirred into a sticky shape. This mixture was then applied to the interdigitated electrode and allowed to dry naturally in an indoor environment, as shown in figure 1*a* [30,31]. After drying, the electrode was placed in a tube furnace and sintered under N_2_ protection at 250°C for 1 h. Subsequently, the Pt/TiO_2_/C sensor electrode was obtained by allowing the electrode to cool to room temperature. The prepared electrode was exposed to a formaldehyde gas environment for gas sensitivity testing, as illustrated in figure 1*b*, and the sensing performance of the material was assessed based on the response strength (*A*).

$$A=\frac{I_{a}-I_{0}}{I_{0}},$$

where *I*_0_ represents the current of the material in the air environment, and *I*_a_ represents the current of the material in the formaldehyde environment [32].

A controlled experimental study was conducted to investigate the impact of Pt and bamboo charcoal addition on the sensing performance of Pt/TiO_2_/C. Three types of materials were prepared using the hydrothermal method with the same concentration ratio of TiO_2_, Pt/TiO_2_ and bamboo charcoal co-modified TiO_2_ (TiO_2_/C). The materials were then fabricated into electrodes, and their sensing performance was compared with that of Pt/TiO_2_/C.

To determine the optimal composite ratio, the quantity of Pt added to the Pt/TiO_2_/C with a 10% Pt modification (the molar ratio of Pt to TiO_2_ is 0.1 : 1) was adjusted. Subsequently, the sensing performance of electrodes fabricated from materials with different Pt modifications, including 2.5% Pt (0.25@Pt, 0.25 times the original amount), 5.0% Pt (0.5@Pt), 20% Pt (2.0@Pt) and the original 10% Pt modification (1.0@Pt), was compared to determine the optimal composite ratio. After selecting the better material, the composite ratio of 0.1 g bamboo charcoal in Pt/TiO_2_/C was varied. The sensing performance of electrodes fabricated from materials with different amounts of bamboo charcoal added, including 0.01 g bamboo charcoal (0.1@C, 0.1 times the original amount), 0.05 g bamboo charcoal (0.5@C), 0.2 g bamboo charcoal (2.0@C) and the original 0.1 g bamboo charcoal (1.0@C), was then compared to determine the optimal composite ratio.

## 3. Results and discussion

### 3.1. Characterization of Pt and bamboo charcoal co-modified TiO_2_

**Figure 2.**
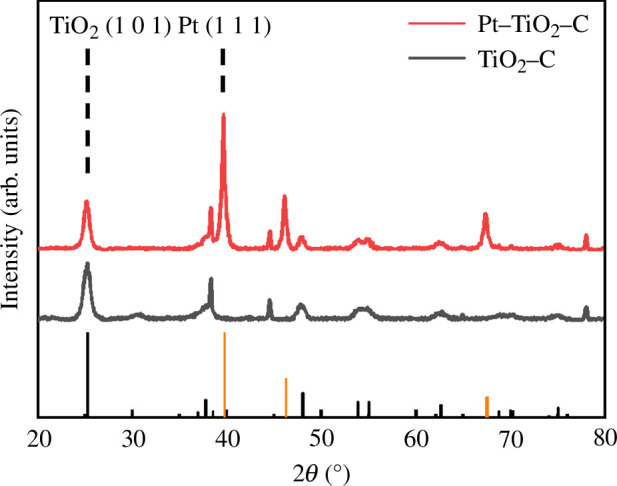
XRD patterns of TiO_2_/C and Pt/TiO_2_/C.

The crystal structure of nano-TiO_2_ was analysed using XRD. Figure 2 shows the XRD patterns of TiO_2_/C and Pt/TiO_2_. The five strong diffraction peaks at 25.28°, 38.34°, 47.82°, 53.96° and 55.06° correspond to the (1 0 1), (0 0 4), (2 0 0), (1 0 5) and (2 1 1) planes of the anatase phase, respectively, indicating the presence of the anatase phase of TiO_2_ [33–36]. The JCPDS card number for TiO_2_ is 71-1167, which confirms these assignments. The particle size of the main body of TiO_2_, calculated from the XRD diffraction peaks, is 12.7 nm, demonstrating the nanoscale nature of the TiO_2_ particles. The pattern of Pt/TiO_2_ shows that the diffraction peaks of Pt were obvious at 39.80°, 46.28° and 67.53°, indicating that Pt has been successfully modified onto the material. The JCPDS card number for Pt is 87-0646, supporting the identification of platinum on the TiO_2_ surface.

**Figure 3.**
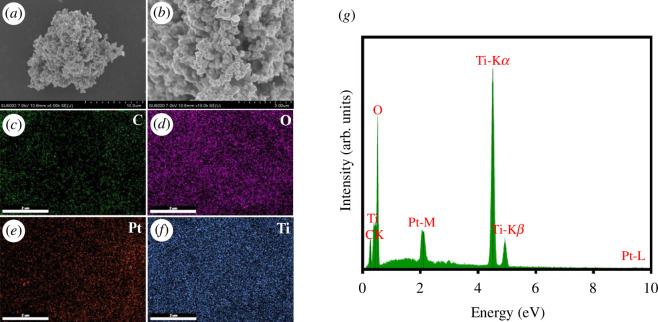
(*a*) Low-magnification and (*b*) high-magnification SEM images of Pt/TiO_2_/C. (*c*) Surface C elements, (*d*) surface O elements, (*e*) surface Pt elements, (*f*) surface Ti elements and (*g*) surface EDS-point EDS mapping of Pt/TiO_2_/C.

The morphology of Pt/TiO_2_/C was investigated by scanning electron microscopy (SEM). As shown in figure 3*a,b*, Pt/TiO_2_/C is composed of numerous small particles with a diameter of 20–30 nm, which belong to typical TiO_2_ particles. Figure 3*c*–*f* shows the energy dispersive spectrometry (EDS) surface scan element distribution for C, O, Pt and Ti in the region of figure 3*b*, respectively. The large amount of O and Ti elements in the figure indicates that the material is TiO_2_, while Pt and C were successfully modified on the surface of the material. Figure 3*g* shows the EDS-point scan result in the region of figure 3*b*, from which C, O, Pt and Ti peaks can be clearly seen. These results indicate that Pt was modified on TiO_2_ and successfully compounded with bamboo carbon.

**Figure 4.**
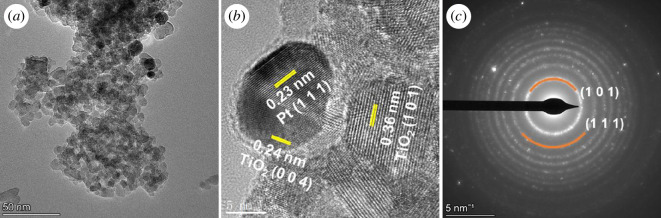
(*a*) TEM, (*b*) HRTEM and (*c*) SAED images of Pt/TiO_2_/C.

The Pt/TiO_2_/C sample was further characterized by TEM. Figure 4*a* shows a low-resolution TEM image of the sample, and the measurements indicate that the particle size of Pt and bamboo charcoal co-modified TiO_2_ was approximately between 6 and 15 nm, which is consistent with the particle size calculated from the XRD characterization mentioned earlier. The representative high-resolution transmission electron microscopy (HRTEM) image in figure 4*b* shows uniform lattice fringes, where the lattice spacing of 0.36 and 0.238 nm corresponds to the (1 0 1) and (0 0 4) planes of anatase TiO_2_, while the lattice spacing of 0.226 nm corresponds to the (1 1 1) planes of Pt [37–39]. Figure 4*c* presents the selected area electron diffraction (SAED) pattern image of the sample, from which the diffraction rings are very clearly visible, indicating the good crystallinity of the sample. The diffraction rings corresponding to (1 0 1) of anatase TiO_2_ and (1 1 1) of Pt are also evident in figure 4*c*.

**Figure 5.**
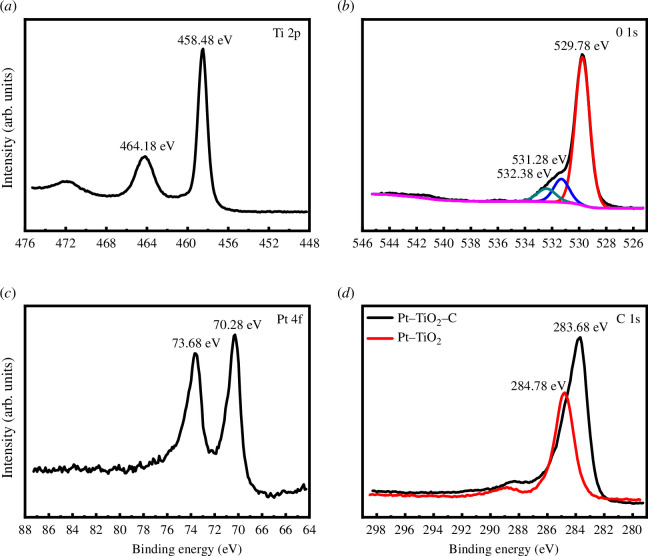
(*a*) Ti 2p XPS spectra, (*b*) O 1s XPS spectra and (*c*) Pt 4f XPS spectra of Pt/TiO_2_/C. (*d*) C 1s XPS spectra of Pt/TiO_2_ and Pt/TiO_2_/C.

The chemical states of Ti, O, Pt and C in TiO_2_/C and Pt/TiO_2_/C were investigated by XPS. For the Pt/TiO_2_/C sample, the binding energies of Ti 2p_3/2_ and Ti 2p_1/2_ are 458.48 and 464.18 eV, respectively, corresponding to the typical Ti^4+^ spectra in TiO_2_, as shown in figure 5*a*. The O 1s spectra of the sample are shown in figure 5*b*. The binding energy band of O 1s can be deconvoluted into three peaks. The highest peak, located at 529.78 eV, usually represents the lattice oxygen in TiO_2_, which is the most common O state in the sample. The peak at 531.28 eV can be ascribed to the surface-bonded OH species related to oxygen vacancy [40]. The smallest peak, located at 532.38 eV, can be attributed to free OH species or the adsorbed O_2_. Figure 5*c* shows the binding energy of Pt 4f for the sample. Two binding energy peaks at 70.28 and 73.68 eV are observed, which belong to Pt 4f_7/2_ and Pt 4f_5/2_ for metallic Pt, respectively. Figure 5*d* shows the binding energy of C 1s for the sample. The addition of bamboo charcoal in the material caused a high peak at 283.68 eV in the spectrum, which also indicates that bamboo charcoal and Pt/TiO_2_ were successfully combined.

### 3.2. The sensing performance of TiO_2_ series samples

**Figure 6.**
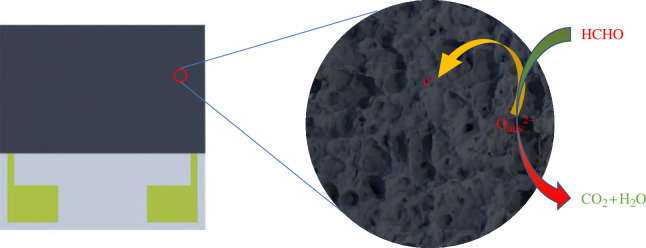
A schematic diagram of the proposed reaction mechanism of the TiO_2_-based sensor in formaldehyde.


Numerous oxygen vacancies exist on the surface of TiO_2_ nanoparticles, which can adsorb O_2_ from the air until reaching saturation. Upon applying a certain voltage to the material for a period of time, the electrons in the nanoparticles react with the adsorbed O_2_ on the material’s surface to form O^2−^, leading to an increase in material resistance that remains stable [41]. Upon exposure of the material surface to formaldehyde gas, it reacts with O^2−^ on the surface, generating water and carbon dioxide, releasing free electrons and reducing the material’s resistance, as demonstrated in figure 6. The reactions can be described by the following equations (3.1)–(3.6):


(3.1) $${\text{O}}_{\text{2gas}}↔{\text{O}}_{\text{2ads}},$$

(3.2) $${\text{O}}_{\text{2ads}}+e^{-}↔{\text{O}}_{\text{2ads}}^{-},$$

(3.3) $${\text{O}}_{\text{2ads}}^{-}+e^{-}↔{\text{2O}}_{\text{ads}}^{-},$$

(3.4) $${\text{O}}_{\text{ads}}^{-}+e^{-}↔{\text{O}}_{\text{ads}}^{2-},$$

(3.5) HCHO(gas)↔HCHO(ads),

(3.6) $$\text{HCHO}\left(\text{ads}\right)+2{\text{O}}_{\text{2ads}}^{-}\to {\text{CO}}_{\text{2}}\text{(gas)}+{\text{H}}_{\text{2}}\text{O(gas)}+\text{4}{\text{e}}^{-}.$$

**Figure 7.**
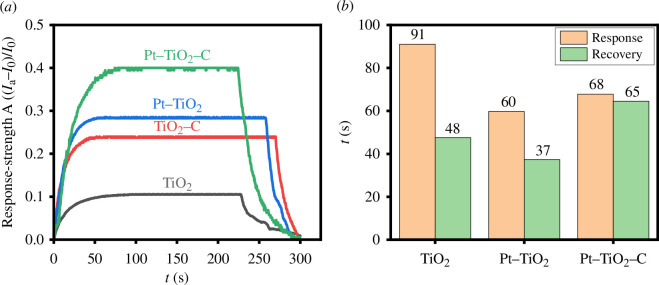
The sensing performance of TiO_2_ series samples. (*a*) The response curves of different electrodes. (*b*) The average response time and average recovery time of different electrodes.

The response curves shown in figure 7*a* were obtained by placing the four electrodes in a 50 ppm formaldehyde environment. The response of the TiO_2_ electrode to 50 ppm formaldehyde was 0.1038. The response of the TiO_2_ electrodes modified with Pt or bamboo charcoal to 50 ppm formaldehyde increased to 0.2866 and 0.2381, respectively. Additionally, the response of the TiO_2_ electrode modified with both Pt and bamboo charcoal to 50 ppm formaldehyde greatly increased to 0.3920. The elevated work function of metallic platinum (Pt) causes electrons from titanium dioxide (TiO_2_) to transfer to platinum. Furthermore, the p-type PtO_2_ will establish a p–n junction with n-type TiO_2_, resulting in the movement of electrons from TiO_2_ to PtO_2_. Both phenomena contribute to the extension of the space-charge layer. According to the sensing mechanism, the expanded space-charge layer in the initial state leads to heightened sensitivity promotion. Moreover, the ultra-small dimensions of TiO_2_ and the ample pores in the matrix provide an elevated specific surface area for the adornment of Pt and PtO_2_, thereby enhancing their functionality [42].

Figure 7*b* shows the average response time and average recovery time of different electrodes to formaldehyde. The response time and average recovery time of the TiO_2_ electrode were 91 and 48 s, respectively. The response time and average recovery time of the Pt/TiO_2_ decreased to 60 and 37 s, respectively. However, the response time and recovery time increased after the addition of bamboo charcoal, which is probably owing to its high porosity and good tubular carbon structure. When formaldehyde comes into contact with the material, it first interacts with the porous surface of bamboo charcoal to adsorb onto the surface, and then interacts with the TiO_2_ surface to generate an electrical signal response. Therefore, the response and recovery time of the material to formaldehyde increase.

The gas-sensing performance of a Pt/TiO_2_-based sensor greatly depends on the amount of Pt used. A series of tests was carried out to discover the effect of Pt dosage on responses. To investigate the effect of Pt content on formaldehyde sensing, the prepared materials were varied by changing the amount of Pt added during their preparation.

**Figure 8.**
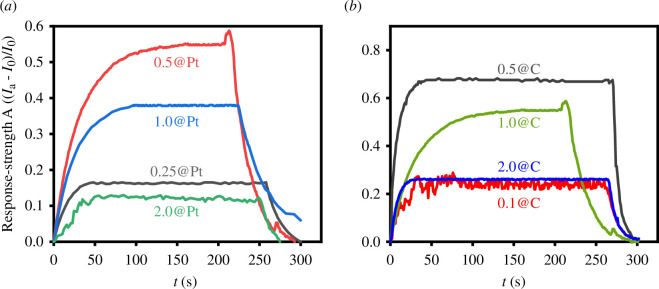
The performance of Pt/TiO_2_/C. (*a*) Different Pt content assisting, relative to the initial proportion of 0.25 times, 0.5 times, 1 times and 2 times. (*b*) Different bamboo content compound, relative to the initial proportion of 0.1 times, 0.5 times, 1 times and 2 times.

The comparison of the response curves for materials with different Pt ratios to formaldehyde is shown in figure 8. Figure 8*a* illustrates the sensing performance of the electrodes with different Pt concentrations for formaldehyde. The sensing performance increased in the order of 2.0@Pt < 0.25@Pt < 1.0@Pt < 0.5@Pt. The material 0.5@Pt exhibited the highest response intensity for formaldehyde, with a response intensity of 0.55. Increasing or decreasing the Pt content would degrade the sensing performance.

In order to improve the sensing performance of materials, the influence of carbon assisting on formaldehyde sensing was further studied. The sensing performance of formaldehyde was investigated by varying the composite ratio of bamboo charcoal in the previously tested 0.5@Pt electrode, as shown in figure 8*b*. From the figure, the sensing performance increased in the order of 0.1@C < 2.0@C < 1.0@C < 0.5@C. The initial experimental results showed that a relatively high composite ratio of bamboo charcoal improved the sensing performance of the electrode. However, reducing the amount of bamboo charcoal further improved the sensing performance. Too much or too little bamboo charcoal led to a decrease in the sensor’s sensitivity to formaldehyde. These results suggest that bamboo charcoal can significantly enhance the sensing performance of the material for formaldehyde, but an optimal amount is required for optimal performance.

**Figure 9.**
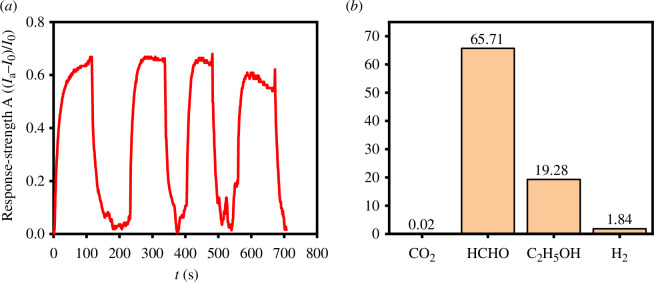
(*a*) The current–time measurements of 0.5@C for HCHO, (*b*) The selectivity of 0.5@C to CO_2_, HCHO, C_2_H_5_OH and H_2_.

The sensing performance of the material 0.5@C was tested for multiple rounds, and the results shown in figure 9*a* were obtained. It can be observed from the figure that after four rounds of cyclic testing, there is no significant change in the response rate and response time of the material, indicating good stability. Furthermore, high selectivity is a crucial characteristic of gas sensors for identifying mixed gases containing target gases, particularly particles with relative physical and chemical properties. In this study, the selectivities of the nano-TiO_2_ samples were tested by exposing them to 1000 ppm of different gases, including carbon dioxide (CO_2_), formaldehyde (HCHO), ethanol (C_2_H_5_OH) and hydrogen (H_2_) at room temperature. As shown in figure 9*b*, the sensor displays responses of 65.71 to HCHO, subsequently 19.28 to C_2_H_5_OH, 1.84 to H_2_ and 0.02 to CO_2_, respectively. The response of the material to HCHO is significantly higher than those of other gases, indicating better selectivity towards formaldehyde.

## 4. Conclusion

In summary, TiO_2_ nanoparticles modified with Pt and bamboo charcoal were successfully prepared using the hydrothermal method. Performance tests of four materials demonstrated that the introduction of Pt and bamboo charcoal enhanced the sensing performance of TiO_2_ for formaldehyde and reduced the response and recovery time of the materials. By varying the ratios of the materials prepared via the hydrothermal method, it was concluded that Pt/TiO_2_/C with a molar ratio of 0.05 : 1 : 1.25 exhibited superior sensing performance and stability for formaldehyde. Furthermore, material 0.5@C demonstrated good stability through multiple rounds of testing in a 50 ppm formaldehyde environment. Lastly, by comparing the response of the material to different gases, it was revealed that the material exhibited better selectivity for formaldehyde. This study introduces a new family of Pt and bamboo charcoal co-modified TiO_2_ materials, providing a novel approach to constructing formaldehyde sensors for detecting low concentrations of formaldehyde at room temperature.

## Data Availability

Experimental data can be accessed from the Dryad Digital Repository [43].
